# Development of the Nurses' Observation Scale for Cognitive Abilities (NOSCA)

**DOI:** 10.5402/2011/895082

**Published:** 2011-07-17

**Authors:** Anke Persoon, Liesbeth Joosten-Weyn Banningh, Wim van de Vrie, Marcel G. M. Olde Rikkert, Theo van Achterberg

**Affiliations:** ^1^Department of Geriatrics, Radboud University Nijmegen Medical Centre, P.O. Box 9101, 6500 HB Nijmegen, The Netherlands; ^2^Department of Medical Psychology, Radboud University Nijmegen Medical Centre, P.O. Box 9101, 6500 HB Nijmegen, The Netherlands; ^3^Nursing Science Section, The Centre for Quality of Care Research, Radboud University Nijmegen Medical Centre, P.O. Box 9101, 6500 HB Nijmegen, The Netherlands

## Abstract

*Background*. To assess a patient's cognitive functioning is an important issue because nurses tailor their nursing interventions to the patient's cognitive abilities. Although some observation scales exist concerning one or more cognitive domains, so far, no scale has been available which assesses cognitive functioning in a comprehensive way. *Objectives*. To develop an observation scale with an accepted level of content validity and which assesses elderly patients' cognitive functioning in a comprehensive way. *Methods*. Delphi technique, a multidisciplinary panel developed the scale by consensus through four Delphi rounds (>70% agreement). The International Classification of Functioning/ICF was used as theoretical framework. *Results*. After the first two Delphi rounds, the panel reached consensus about 8 cognitive domains and 17 sub domains. After two other rounds, 39 items were selected, divided over 8 domains and 17 sub domains. *Discussion*. The Nurses' Observation Scale Cognitive Abilities (NOSCA) was successfully designed. The content validity of the scale is high because the scale sufficiently represents the concept of cognitive functioning: the experts reached a consensus of 70% or higher on all domains and items included; and no domains or items were lacking. As a next step, the psychometric qualities of the NOSCA will have to be tested.

## 1. Background

The vulnerability of elderly hospital patients is characterised by simultaneously occurring somatic, psychological, and social problems, which may result in problems in cognitive functioning, mood, behaviour, activities of daily life, and, consequently, in declining quality of life. Determination of an individual's specific cognitive status is important for two reasons. First, the choices of nursing interventions are substantially influenced by the patient's cognitive abilities. The patient's cognitive abilities determine the provision of nursing care to a large extent as they influence communication, the support to be given in daily life activities, the recognition and treatment of other nursing problems (e.g., pain, behavioural problems), and discharge policy [1–3]. The nurse's approach to individual patients is also largely influenced by the type of cognitive problem. In case of memory problems, for example, information is repeated or written down; in case of problems in sustaining attention, a quiet environment is offered; and in case of executive problems, information is kept simple. Second, facilitation of medical diagnosis is another reason for determining cognitive status. Neuropsychiatric disorders show specific types of cognitive dysfunctioning. For example, memory problems are often the first sign of Alzheimer's disease, whereas loss of awareness may indicate frontotemporal dementia, and hallucinations suggest delirium and dementia with Lewy bodies. 

### 1.1. Cognitive Decline

Cognitive functioning is a term used to address the wide area of human information processing. The concept of cognitive functioning is operationalized by breaking it down into several cognitive domains. However, there is no uniform way to classify the cognitive domains; different authors organise the cognitive domains in different ways [4–6]. Cognitive decline may occur due to age-related factors, depression, delirium, dementia, and in combination with serious somatic health problems. Recognition of delirium is most important because of its high incidence rate and reversible character, for example, by means of the Confusion Assessment Method (CAM; [7]) and the Delirium Observation Scale (DOS; [8]). However, comprehensive cognitive assessment still needs to be carried out as other types of brain dysfunction might occur, such as dementia or brain injury. A diagnosis is then based on medical history, neurological assessment, neuropsychological assessment, neuroimaging, complaints of patient, and behavioural symptoms.

### 1.2. Observation of Cognitive Abilities

Direct observation of the patient's cognitively mediated abilities complements cognitive assessment [2, 9]. Daily observation of the patient covers 24 hours a day and may last for several days. It is based on informal interactions between the patient and nurse, for example, when taking a bath, having breakfast, during transfers, or when interacting with other patients. The observation is not threatening, burdensome, or stressful for patients. The patient's cooperation is not required, and observation can be conducted even when patients are too ill to undergo neuropsychological testing. Unlike testing, which assesses cognitive abilities under optimal experimental conditions, direct observation assesses a person's cognitive abilities in daily life. Results of daily observation are therefore of high ecological validity as they are linked to the natural setting of daily life and do not depend on one specific test moment [9, 10]. Observation may improve the reliability of cognitive assessment if serial observations are recorded on several consecutive days. Furthermore, observation fits very well into the nursing practice, because information is directly accessible during patient care encounters.

One problem with direct observation is its standardisation [6]. Yet, well-validated observation scales are rather scarce, although some good examples exist in geriatrics, for example, for depression, pain, agitation, dementia, and as mentioned above, for delirium. We searched the literature for direct observation scales for cognition abilities, excluding, however, delirium screening instruments [18]. The scales identified by means of this search are presented in Table 1. We found instruments which assess cognitive functioning in a limited way (not divided into subscales of cognitive domains: e.g., the OLD), facilitate just one or two cognitive domains (e.g., the NOSGER), or are too specific for nurses (the A-one). Furthermore, many of them also involved issues such as mood or behavioural problems (e.g., MOSES and NOSGER). We therefore concluded that there is no valid observation scale available for nurses to comprehensively assess cognitive functioning in relation to possible dementia or brain injury [18]. 

In daily practice, this means that individual nurses observe cognitive functioning in patients in a nonmethodological way. This undermines the reliability and validity of the information obtained, as demonstrated in two studies on Dutch geriatric hospital wards [3, 19]. The nurses in these studies assessed different cognitive domains per patient, aimed at different goals. In addition, only moderate agreement was reached between these nurses.

## 2. Objectives

The aim of this study was to develop an observation scale with an acceptable level of content validity which assesses elderly patients' cognitive abilities in a comprehensive manner, including a wide range of cognitive domains. The resulting Nurses Observation Scale for Cognitive Abilities (NOSCA) had to fulfil certain preconditions:

1. it should be suitable for observation of the population of geriatric patients admitted in acute care hospitals;
2. it should structure around-the-clock observations by nurses and possibly include all interactions naturally occurring between patient and nurse (e.g., bathing, meal times or transfers, small talk, informing, educating);
3. it should serve goals important in daily practice, that is, it should enable tailoring to individual (nursing) care plans, contribute to the diagnostic process, or facilitate further neuropsychological examination.

## 3. Methods

### 3.1. Content Validity

The works by Haynes [20], Polit and Beck [21], Streiner and Norman [22], and Foreman et al. [5] on the development of (cognitive) measurement scales constituted the point of departure for our study. They all underpin the necessity to explicitly decide on the aim and context of the measurement instrument to be developed. Therefore, we clearly formulated the preconditions, see section Aims. Most important in designing a measurement instrument is maximising content validity. Content validity concerns the conceptualisation of the content and the degree to which the scale represents the concept. Content validity is the degree to which an instrument has an appropriate number of items for the construct being measured [21]. As no uniform way to classify cognitive domains was found in the literature, a panel of experts was required to evaluate the content validity of the scale developed [21]. We obtained a written judgment from the experts concerning the NOSCA construct and items by means of the Delphi technique. In several rounds during which experts' preferences were integrated, consensus was achieved concerning the NOSCA construct: the cognitive domains (Phase 1) and items (Phase 2). In Phase 3, we conducted a preliminary test to assess its feasibility.

### 3.2. Theoretical Framework

We proceeded from the definition of cognitive function as cited by a nursing protocol found in the literature [5], which was based on the work of Lezak: “Cognitive functioning encompasses the processes by which an individual perceives, registers, stores, retrieves and uses information” [23]. As the scale had to have a firm theoretical basis and no consensus was found in the geriatric, psychiatric, or neuropsychological field, we selected the more general health-based International Classification of Functioning (ICF) [24]. The ICF includes a classification system of functions, in which Chapter 1 states the Mental Functions. Some of these mental functions relate to cognitive functions.

To enhance reliability, items had to be written for observable patient activities or behaviour, noticeable for all observers, so that bias in the observer's interpretation would be minimised. As the patient's cognitive functioning varies depending on the moment of the day, type of activity, or interaction with others, we preferred more than one observation. The observation should take place twice a day during two consecutive days in order to obtain sufficiently reliable outcome and should also account for inter-daily variation. To improve reliability, it was required that the scale be completed at the end of every shift.

### 3.3. Multidisciplinary Panel

The multidisciplinary panel consisted of 16 experts and was composed of geriatric nurses (2x), advanced nurses in geriatrics (5x), one nurse lecturer in geriatrics, geriatricians (3x), neuropsychologists (4x), and one occupational therapist (see Table 2). All of them had many years of clinical practice experience with elderly people and were in some way experts in assessing cognitive functioning. Of the advanced nurses, two had additional experience in developing a measurement instrument for assessing elderly people, two nurses were familiar with the ICF, and four of them had published (internationally) on cognitive functioning. Three out of the four neuropsychologists had experience in developing a measurement instrument and had published in international journals. The geriatricians were selected because of their clinical expertise and the research they had conducted. Of the originally invited experts, only one refused because the Delphi rounds would be too time-consuming. All the experts received information about the objective of the observation scale, the setting, and the theoretical framework.

**Phase 1: Domains Included**

In this phase, the construction of the scale was established by means of the 1st and 2nd Delphi rounds. The aim was to determine the cognitive domains to be included in the scale. In the 1st Delphi round, nine of the cognitive domains mentioned in Chapter 1 of the ICF were presented to the panel for possible inclusion in the observation scale. This information also included the ICF definition of the cognitive domain and 19 ICF subdomains. Furthermore, all functions as described in this chapter were presented, so experts were able to judge the representativeness of the nine domains proposed. The experts were requested to respond on the following: the relevance of the ICF domain (also including the formulation of the ICF label and ICF definition) and the relevance of the subdomain (also including the formulation of the ICF label and ICF definition), representing all important cognitive domains. It was required that at least 70% of the experts agree that a proposed cognitive domain should be included. Suggestions from individual experts to rephrase or alter certain parts or to include new (sub)domains were presented to the panel in the second round.

**Phase 2: Item Selection**

In this phase, the items of the scale were determined by means of the 3rd and 4th Delphi rounds. Central in this phase was the question as to whether an item was relevant to a domain or subdomain and whether the items sufficiently represented the domain. The items presented to the panel were mainly derived from other observation scales (see Table 1). This resulted in a total of 173 items. All the items were reformulated using the same sentence structure, for example, “The patient is able to locate his/her own bed”. Furthermore, we placed the items into matching domains and subdomains.

In the 3rd round, we presented the panel with the 173 items, divided across the domains and subdomains. The experts were requested to respond on the following: the relevance of the item and the priority of the item, representing all important items. Items which were relevant according to 70% of the panel were listed. Then, the items approved were checked for interdependency. When it turned out that too many items were approved, we selected the best observable behaviour or activity. Subsequently, the approved items were checked again in order to assess whether they differentiated from items in the other cognitive domains. If they did not, we selected the items which seemed to be most appropriate for certain domains. Finally, the suggestions made by the experts concerning the rephrasing of items or inclusion of new items were incorporated.

In the 4th Delphi round, items which were still under discussion, newly suggested, or located in another domain or subdomain were reevaluated by the experts.

**Phase 3: Pretest**

In this phase, the feasibility of the observation scale was tested in a small study. Over a period of two weeks, nurses from one geriatric ward were asked to complete the observation scale on the basis of their observation of a single patient during their shift. The nurses were asked for comments on the instructions and on the observation scale. Some of these comments were subsequently processed. After seven nurses had provided their comments, no further suggestions for adjustments were made.

## 4. Results

**Phase 1: Domains Included**

In the first two Delphi rounds, the response from the 16 panel members was 100%.  Table 3 presents an overview of the panel's acceptance of the domains and subdomains and the suggestions they made. After the 1st Delphi round, all nine proposed domains were accepted (>80% agreement), as well as 16 out of the 19 proposed subdomains. Nine suggestions for including new subdomains were made. The response on the Consciousness domain showed insufficient consensus: although 81% of the experts saw this domain as part of the cognitive function scale, several experts also suggested rephrasing because consciousness is only a prerequisite for cognitive functioning and should therefore have another position in the observation scale compared to the other cognitive domains. We presented this suggestion to the panel in the second Delphi round.

After the 2nd Delphi round, five out of the nine newly suggested subdomains were accepted by the panel (>80% agreement), see Table 3. There was 81% agreement that Consciousness should have a distinct place in the observation scale. Next, as suggested by experts in the first round, the subdomain of Content of Thoughts was added to the 2nd round, and although 81% of the panel agreed to include this subdomain, 64% wished to rephrase the items concerned. The panel was therefore consulted by email on the question of whether or not these two subdomains should be included. Thirteen out of the 16 experts responded, and it was concluded that the subdomain of Dividing Attention should not be included (56% agreed). It was also concluded that the subdomain of Content of Thoughts should be reformulated (85% agreed).

In sum, after two Delphi rounds and one follow-up email, the panel reached consensus on the construct of cognitive function by means of 8 domains and 17 subdomains (see Table 4). Furthermore, the domain of Consciousness (consisting of two subdomains) was considered a prerequisite for cognitive functioning, which should therefore be assessed beforehand.

**Phase 2: Item Selection**

In the 3rd and 4th Delphi rounds, 15 and 16 out of the 16 panel members responded, respectively. After the 3rd round, it turned out that 58 of the 173 items were deemed to be relevant (>70% agreement). We then checked these 58 items for interdependency. 16 items were excluded, which left us with a selection of 42 items. Although these 42 items were accepted for the observation scale, the experts suggested rephrasing of seven items and relocating one item to another domain. Three new items were suggested. All in all, in the 4th round, 45 items were presented to the panel of which eleven were to be judged again on relevance, rephrasing, and representativeness. After the 4th round, the panel accepted nine out of the eleven items as being relevant to the new observation scale and well-formulated (>70% agreement).

In sum, after the 3rd and 4th rounds, the observation scale consisted of 39 items, divided across 8 domains and 17 subdomains, preceded by 4 items for the domain of Consciousness as a condition for cognitive functioning (see Table 5).

**Please Read the Following Information about the NOSCA**

**Aim:**

- With this observation list, nurses can gain an impression of whether a patient has cognitive problems and if so, in which domains.
- These observations will be used to (a) contribute to the diagnostics at the multidisciplinary meeting and (b) to help determine the nursing interventions (approach form, information, family education).

**Instructions:**

- Before starting your shift, read the NOSCA items so that you can make targeted observations and if necessary, induce behaviour (e.g., start a conversation, read a text, get dressed, etc.).
- Create the most optimal conditions for the patient (glasses on, hearing aid working).
- Make observations over a period of two consecutive days, in the day shifts and evening shifts. This will lead to four completed forms per patients. Research has shown that more than four observation periods do not lead to better information.
- Record the observations per shift, so that the report is as reliable as possible.

**Filling in the Form:**

- Put a circle around the correct answer in accordance with your observations during one shift. “never” means that the behaviour did not occur during your whole shift. “Repeatedly” means that the behaviour occurred repeatedly during your shift.
- Put a circle around the question mark “?” if the behaviour could not be observed because the situation did not arise (e.g., because the patient did not read anything). Also put a circle around the question mark “?” if the patient could not display certain behaviour (e.g., the patient could not put on his/her clothes in the correct order, because he/she cannot dress independently due to a physical disability).

**Drawing a Conclusion:**

- The observations over four shifts lead to one conclusion. Calculate the average score per subscale, representing a cognitive domain, and note it on the summary sheet (range 0–3 points). The NOSCA overall score is calculated by the sum of the eight domains (range 0–24 points).
- Norm values of the subscales: lower scores indicate less cognitive abilities:
  - 3 means that no problems were observed;
  - 2 means that problems sometimes arose;
  - 1 means that problems usually arose;
  - 0 means that problems arose repeatedly.

**Phase 3: Pretest**

The feasibility of the concept of the observation scale was tested seven times consecutively. Five times improvements were made for the sake of clarity; the last two tests showed no need for adjustments. In general, nurses had no difficulties filling in the form and it took them only a few minutes per patient per shift. The instructions were changed several times, and the layout of the items was improved, resulting in a more concise and clear text.

## 5. Conclusion

The object of this study was to develop an observation scale in which cognitive abilities are assessed in a comprehensive manner, and which has an acceptable level of content validity. We succeeded in designing the Nurses' Observation Scale Cognitive Abilities (NOSCA), in which cognitive functioning is classified into eight domains, 17 subdomains, and 39 items, preceded by 4 items on the domain of Consciousness. The content validity of the NOSCA, as a measurement of the degree to which the scale represents the concept of cognitive functioning, is high for two reasons. First, the minimum agreement between the panel members was 70%, and often higher on certain domains and items. Second, after the fourth Delphi round, consensus was reached that no domains or items were lacking. In the preceding Delphi rounds, the panel had made suggestions for including new domains and items, and some were accepted in the next Delphi round. The strength of our procedure was that the panel members represented four disciplines (nursing, neuropsychology, geriatrics and speech, therapy). Thus, the quality of the panel was enhanced because each discipline with its specific focus and knowledge of the concept of cognitive functioning provided specific input for the observation scale. An equally strong point of the NOSCA and its development is that the scale is based on the ICF's theoretical framework. Although the ICF is not a cognitive concept but a general classification of functioning, it proved to be very suitable for the purpose of our study and probably will increase acceptability in the field. 

The method we used had only one drawback: in designing the NOSCA, we were required, due to lack of consensus on the concept of cognitive functioning in the literature, to develop consensus by means of the Delphi technique. The disadvantage of this technique is that consensus depends on the current state of the art in the professional disciplines and that, through the years, the state of art will further develop. Particularly as a result of the new technique of neuroimaging, it is expected that the knowledge of brain functions will increase in the coming years, and consequently the observation scale may have to be revised after some time.

Now that the content validity has been described, the psychometric qualities of the NOSCA will have to be tested. Internal consistency, interrater reliability, and intrarater reliability will be examined, as well as construct validity. For the latter, results of the NOSCA will be related to clinical diagnoses, severity of dementia, and results of neuropsychological tests. Discriminant validity will be studied by comparing the results of the NOSCA with scores on depression. 

Expecting positive results on the psychometric qualities, we are convinced that the implications of the NOSCA for the nursing practice will prove important. Nurses will be better equipped to tailor interventions to patients' cognitive abilities. Furthermore, behavioural observation within a clinical environment provides invaluable information regarding underlying cognitive function [25]. The value of observation of daily behaviour for assessing cognitive functioning was the reason for Miller et al. to develop the BATCH (after we had finished our literature search). This is an assessment tool to record observations of patients' daily functioning under subheading that reflect cognitive domains [25]. They were interested in an observation scale for patients in a psychiatric setting (mean age 50 years) who refuse or cannot undertake cognitive assessments. The BATCH covers ten cognitive domains and comprises 60 items. No information about item selection is given. The BATCH may well be interesting if more data are collected on validity, reliability, distinction between the subscale scores and if the scale were to be applied in a geriatric patient group. For geriatric patients, several methods for assessing their cognitive functioning need to be combined, and daily observation of cognitive functioning remains important because of its high ecological value. 

We expect that the NOSCA will be useful at hospital medical and surgical wards as well, because the items all cover behaviour which is easy to observe, and thus the scoring does not require specific geriatric expertise or knowledge. In other settings, such as home care and homes for the elderly, it will also be useful in assessing cognitive functioning. For these different settings, separate validation studies will be required. 

For the time being, we recommend that nurses closely observe elderly patients and report cognitive functioning and dysfunctioning, and associated behaviour. Improving these systematic observations will enhance the ability of nurses to truly tailor their interventions to the patients' abilities. The NOSCA is a promising tool that will help nurses to perform the challenging observations of cognitive functioning, and thereby improve the quality of care provided to the fast growing number of older patients.

##  Funding

This research received no specific grant from any funding agency in public, commercial, or not-for-profit sectors.

##  Conflict of Interests

No conflict of interests has been declared by the authors.

## Figures and Tables

**Table 1:** Scales with a focus on direct observation of cognitive function (delirium screening scales excluded).

Scale^(1)^	Authors	Setting	Population	Subscales concerning cognition^(2)^	Cognitive domains^(3)^ (*n*)
A-one	Arnadottir, 1990 [11]	Hospital	Trauma	Motor apraxia, ideational apraxia, body neglect, somatoagnosia, spatial neglect, abnormal tone, perseveration, organisation, sequencing, sensory and expressive aphasia, dysarthria, jargon aphasia, paraphasia, perseveration, anomia	5
Bans-S	Volicer et al., 1987 [12]	Nursing home	Dementia	Speech	1
CPS (MDS/RAI)	Morris et al., 1994 [13]	Nursing home	Dementia	Comatose, short-term memory, decision making, making self understood	4
GIP	Verstraten, 1988 [14]	Nursing home	Elderly	Consciousness, incoherence, memory disorders, disoriented behaviour, aimless repetitive behaviour, suspiciousness	5
MOSES	Helmes et al., 1987 [15]	Nursing home	Elderly	Disorientated behaviour	1
NOSGER	Spiegel et al., 1991 [16]	Hospital	Elderly	Memory	1
OLD	Hopman-Rock et al., 2001 [17]	General practice	Dementia	Forgetfulness, repetition, language, understanding, orientation.	5

^(1)^Scales: A-one: Árnadóttir OT-ADL Neurobehavioral Evaluation.

Bans-S: Bedford Alzheimer Nursing Scale.

CPS: Cognitive Performance Scale, subscale from Minimum Data Set (MDS) and part of the National Residential assessment Instrument for nursing homes RAI.

GIP: Nurses' Behavioural Rating Scale for Geriatric Inpatients.

MOSES: Multidimensional Observation Scale for Elderly subjects.

NOSGER: Nurses' observations scale for geriatric patients.

OLD: Observation List for early signs of Dementia.

^(2)^Titles of the cognitive subscales. The noncognitive subscales are not listed.

^(3)^Cognitive domains of the subscales classified by the seven domains as described by Foreman et al. [5]: alertness/consciousness, attention, memory, thinking, perception, psychomotor behaviour, higher cognitive functions.

**Table 2:** Characteristics of experts in the panel (*n* = 16).

Expert	Discipline^(1)^	Education^(2)^	Field	Setting^(3)^	Publications^(4)^
1	N	RN	Geriatrics	UH	—
2	N	MScN	Rehabilitation	UH	D
3	N	PhD, MScN	Geriatrics	Univ	I
4	N	MANP	Psychiatry	Psy	D
5	N	RN	Ger	TH	—
6	N	MScN	Psychiatry	UH	I
7	N	MScN	Geriatrics	TH	D
8	N	MScN	Geriatrics	TH	D
9	NP	PhD	Geriatrics	TH	I
10	NP	PhD	Rehabilitation	Univ	I
11	NP	PhD	Geriatrics	Univ	I
12	NP	PhD	Geriatrics	Univ	I
13	M	MD	Geriatrics	UH	—
14	M	MD	Psychiatry	Psy	—
15	M	MD	Geriatrics	TH	I
16	OCC	Occ	Rehabilitation	Reh	—

^(1)^Discipline: N = nursing, NP = neuropsychology; M = medicine, Occ = occupational therapy.

^(2)^Education: GN = geriatric registered nurse, PhD = doctor of philosophy, MScN = master of science in nursing, MANP = NP = master of advanced nursing, MD = medical doctor.

^(3)^Setting: UH = university hospital, Univ = University, Psy = psychiatric hospital, TH = teaching hospital, Rev = rehabilitation centre.

^(4)^Publications: D = Dutch publications, I = international publications.

**Table 3:** The construct of cognitive functioning organised into domains as judged by experts (*n* = 16).

Proposed domains	1st Delphi round				2nd Delphi round			Inclusion
	Accepted* (%)	Proposed subdomain	Accepted (%)	Formula-tion OK (%)	Proposed domains/subdo-mains + rephrasing	Accepted (%)	Formula-tion OK (%)	Accepted
Consciousness (b110)	82	Quality of consciousn. (b1102)	63	31	Deleted			no
					State of conscious. (b1100)	81	70	yes*
					Continuity of conscious. (b1101)	81	46	yes*

Attention (b140)	82	Sustaining attention (b1400)	94	67				yes
		Shifting of attention (b1401)	88	79				yes
		Dividing attention (b1402)	75	75		56		no

Perception (b156)	88	(b1561)	88	79				yes
					(b1560)	56	78	no
					(b1565)	44	58	no

Orientation (b114)	100	Orientation to time (b1140)	100	94				yes
		Orientation to place (b1141)	100	100				yes
		Orientation to person (b1142)	100	94				yes

Memory (b144)	100	Short-term memory (b1440)	88	57	Working memory			yes
		Long-term memory (b1441)	94	73				yes
					Retrieval of old information (b1442.0)	62	80	no
					Storage and retrieval of new information (b1442.1)	56	89	no

Thoughts (b160)	94	Pace of thought (b1600)	82	85				yes
		Form of thought (b1601)	75	92				yes
		Control of thought (b1603)	44	100	Deleted			no
					Content of thoughts (b1602)	88	64	yes

Higher level of cognitive function (b164)	82	Cognitive flexibility (b1643)	63	70	Deleted			no
					Organisation and planning (b1641)	94	100	yes
		Insight (1644)	75	71				yes
		Judgment (b1645)	82	71	Deleted			No
					Self-regulation (b1648)	75	92	yes

Language (b167)	100	Reception of language (b1670)	88	67				yes
		Expression of language (b1671)	94	75				yes

Mental function of sequencing complex movement (b176)	88	—	—	45	label rephrased: Praxis	88	70	yes

*Consciousness is perceived as a prerequisite for cognitive functioning and therefore should have another position in the observation scale.

**Table 4:** Construct and number of items of the NOSCA.

Domain	ICF code	Subdomain	ICF code	*N* items	*N* items
Consciousness*	*b110*	*State of consciousness *	*b1100*	*4*	*2*
		*Continuity of consciousn. *	*b1101*		*2*

Attention	b140	Sustaining attention	b1440	4	2
		Shifting of attention	b1401		2

Perception	b156	Visual perception	b1561	2	2

Orientation	b114	Orientation to person	b1142	6	2
		Orientation to place	b1141		2
		Orientation to time	b1140		2

Memory	b144	Short-term memory	b1440	6	3
		Long-term memory	b1441		3

Thoughts	b160	Pace of thought	b1600	5	1
		Form of thought	b1601		2
		Content of thought	b1602		2

Higher cognitive function	b164	Organisation and planning	b1641	7	2
		Insight	b1644		3
		Self-regulation	b1648		2

Language	b167	Reception of language	b1670	6	2
		Expression of language	b1671		4

Praxis	b176	Praxis	b176	3	3

*Consciousness is a prerequisite, as such it is no part of the observation scale, but a condition to be assessed before application of the observation scale.

**Table:** (a) Consciousness ^*ICF*-*code* *b*110^

The patient …		1	0
A	… responds to being spoken to during the day.	Yes	No
B	… has to be shaken awake during the day or evening if you want to communicate with him/her.	No	Yes
C	… falls asleep when no activities are going on.	No	Yes
D	… dozes off during a conversation or activity	No	Yes

Total Consciousness: … points/number of answers = … points.

Note: if any of the above items are scored in the right-hand column, then the results of the observations below must be interpreted with cautin, because the outcomes might change when consciousness is restored.

**Table:** (b) Attention ^*ICF*-*code* *b*140^

The patient …		3	2	1	0	—

1	… loses the thread of the conversation (e.g., when giving long answers).	Never	Sometimes	Usually	Repeatedly	?
2	… stops with the current activity if someone walks by or if he/she hears another voice.	Never	Sometimes	Usually	Repeatedly	?
3	… can easily switch to a different topic of conversation.	Repeatedly	Usually	Sometimes	Never	?
4	… can easily switch to a different activity.	Repeatedly	Usually	Sometimes	Never	?

Total Attention: … points/number of answers = … points.

**Table:** (c) Visual Perception ^*ICF*-*code* *b*156^

The patient …		3	2	1	0	—

5	… recognizes an object and knows what it is (e.g., a comb to comb his/her hair, a toilet to relieve him/herself).	Repeatedly	Usually	Sometimes	Never	?
6	… mistakes an object for something else (e.g., pattern in the curtains for an animal).	Never	Sometimes	Usually	Repeatedly	?

Total Visual Perception: … points/number of answers = … points.

**Table:** (d) Orientation ^*ICF*-*code* *b*114^

The patient …		3	2	1	0	—

7	… is able to locate his/her own bed.	Repeatedly	Usually	Sometimes	Never	?
8	… thinks that he/she is at home or somewhere else.	Never	Sometimes	Usually	Repeatedly	?
9	… recognizes other patients and/or staff..	Repeatedly	Usually	Sometimes	Never	?
10	… recognizes family and/or friends..	Repeatedly	Usually	Sometimes	Never	?
11	… knows whether it is morning, evening or night.	Repeatedly	Usually	Sometimes	Never	?
12	… knows what time it is.	Repeatedly	Usually	Sometimes	Never	?

Total Orientation: … points/number of answers = … points.

**Table:** (e) Memory ^*ICF*-*code* *b*144^

The patient …		3	2	1	0	—

13	… cannot remember what has just been said.	Never	Sometimes	Usually	Repeatedly	?
14	… cannot remember where he/she has just left something.	Never	Sometimes	Usually	Repeatedly	?
15	… can remember the task or instruction during the ADL activities.	Repeatedly	Usually	Sometimes	Never	?
16	… can remember appointments made today or yesterday.	Repeatedly	Usually	Sometimes	Never	?
17	… is able to find an object or piece of clothing that he/she has tidied up.	Repeatedly	Usually	Sometimes	Never	?
18	… knows whether or not objects belong to him/her.	Repeatedly	Usually	Sometimes	Never	?

Total Memory: … points/number of answers = … points.

**Table:** (f) Thoughts ^*ICF*-*code* *b*160^

The patient…		3	2	1	0	—

19	… responds very slowly to a question and/or instruction.	Never	Sometimes	Usually	Repeatedly	?
20	… gives answers that are relevant to the question.	Repeatedly	Usually	Sometimes	Never	?
21	… switches from one subject to another.	Never	Sometimes	Usually	Repeatedly	?
22	… has unrealistic thoughts (e.g., says that he/she does not have any money or clothes, but does really).	Never	Sometimes	Usually	Repeatedly	?
23	… is distrustful of others (e.g., does not dare to take his/her medicine; says that people are “listening”, etc.).	Never	Sometimes	Usually	Repeatedly	?

Total Thoughts: … points/number of answers = … points.

**Table:** (g) Higher cognitive functions ^*ICF*-*code* *b*164^

The patient…		3	2	1	0	—

24	… can oversee where to start an activity (e.g., collects all the necessary articles together before going to wash)	Repeatedly	Usually	Sometimes	Never	?
25	… works efficiently and systematically.	Repeatedly	Usually	Sometimes	Never	?
26	… asks questions about his/her illness.	Never	Sometimes	Usually	Repeatedly	?
27	… says that he/she is able to do something although it is clear that they cannot (e.g., walk without the rollator).	Never	Sometimes	Usually	Repeatedly	?
28	… says that there is nothing wrong with him/her although there clearly is.	Repeatedly	Usually	Sometimes	Never	?
29	… undertakes activities on his/her own initiative (e.g., starting a conversation, going for a walk)	Repeatedly	Usually	Sometimes	Never	?
30	… keeps on repeating an action that is not necessary (e.g., keeps on spreading a slice of bread, keeps on drying his/her arm).	Never	Sometimes	Usually	Repeatedly	?

Total Thoughts: … points/number of answers = … points.

**Table:** (h) Language ^*ICF*-*code* *b*167^

The patient…		3	2	1	0	—

31	… understands directions and/or instructions.	Repeatedly	Usually	Sometimes	Never	?
32	… reads something and can show that he/she has understood what is says (e.g., a wrapper, a folder).	Repeatedly	Usually	Sometimes	Never	?
33	… has to search for words.	Never	Sometimes	Usually	Repeatedly	?
34	… uses vague terms in conversation (e.g., “You know” or “thingy”).	Never	Sometimes	Usually	Repeatedly	?
35	… calls something by the wrong name (e.g., says vase instead of bread, lamp instead of table).	Never	Sometimes	Usually	Repeatedly	?
36	… is able to make clear what he/she wants.	Repeatedly	Usually	Sometimes	Never	?

Total Language: … points/number of answers = … points.

**Table:** (i) Praxis ^*ICF*-*code* *b*176^

The patient …		3	2	1	0	—

37	… does the ADL activities in the correct order (e.g., first takes off pay pyjamas, then gets dressed; first wets the flannel, than washes face).	Repeatedly	Usually	Sometimes	Never	?
38	… puts on clothes in the correct manner (e.g., not back-to-front, or inside-out).	Repeatedly	Usually	Sometimes	Never	?
39	… uses the items in the correct manner (e.g., is able to comb his/her hair with a comb, is able to eat with a fork).	Repeatedly	Usually	Sometimes	Never	?

Total Praxis: … points/number of answers = … points.
